# An evaluation of the Chemispek multichannel analyser

**DOI:** 10.1155/S1463924681000370

**Published:** 1981

**Authors:** R. W. Logan, A. K. Tweedie, Evelyn M. Aitchison, Elizabeth C. Jamieson

**Affiliations:** Department of Biochemistry Yorkhill Children's and Maternity Hospitals Glasgow UK


reporting errors. An increased computer facility would be particularly useful for laboratories which do not have a sophisticated data-processing system. In its present form, the output of the calculator can be fed directly into data processing systems in current use in clinical biochemistry.

### Conclusions

The Vitatron PA800 is a reliable instrument which produces accurate results on small volumes of serum with minimum wastage of reagents. It is equally suitable for the analysis of large batches of samples for the more commonly requested clinical analyses and for processing small numbers of samples, whether for more specialised analyses or in a laboratory with a small workload. This versatility should enable the instrument to fulfil a useful role in most clinical laboratories.

ACKNOWLEDGEMENTS

The authors would like to thank Mr. Ian Martin for performing the statistical analysis of the precision data, the DHSS and MSE Scientific Instruments, who jointly financed the Evaluation and Mrs. Linda Holmes for secretarial assistance.

REFERENCES

[1] Broughton, P. M. G., Gowenlock, A. H., McCormack, J. J., and Neill, D. W. (1974). Revised scheme for the evaluation of automatic instruments in Clinical Chemistry. *Ann. Clin. Biochem.* **11**, 207-218.

[2] Patel, Shanta and O'Gorman P. (1975). Assessment of a new enzyme reaction rate analyser, the Vitatron AKES. *Clin. Chim. Acta* **60**, 249-258.

[3] McQueen, M. J., King, J. (1957). Evaluation of the Vitatron Automatic Kinetic Enzyme System. *Clin. Chim. Acta* **64**, 155-164.

[4] Young, D. S., and Gochman, N. (1972). Methods for assuring quality data from continous flow analysers. *Stand. Methods in Clin. Chem.* **7**, 293-304.

[5] The Committee on Enzymes of the Scandinavian Society for Clinical Chemistry and Clinical Physiology (1974). Recommended methods for the determination of four enzymes in blood. *Scand. J. Clin. Lab. Invest.* **33**, 291-305.

[6] Jendrassik, L. and Gróf, P. (1938). *Vereinfachte photometrische Methoden zur Bestimmung des Blutbilirubins. Biochem Z.* **297**, 81-89.

[7] Michaelsson M., Nosslin, B. and Sjölin, S. (1965). Plasma bilirubin determination in the newborn infant. *Pediatrics* **35**, 925-931.

[8] Billing, Barbara, Haslam, Ruth and Wald, N. (1971). Bilirubin standards and the determination of bilirubin by manual and Technicon AutoAnalyser methods. *Ann. Clin. Biochem.* **8**, 21-30.

[9] Röschlau, P., Bernt, E. and Gruber, W. (1974). Enzymatic determination of total cholesterol in serum. *Z. Klin. Chem. Klin. Biochem.* **12**, 403-407.

[10] Trinder, P., (1969). Determination of glucose in blood using glucose oxidase with an alternative oxygen acceptor. *Ann. Clin. Biochem.* **6**, 24-27.

[11] Nobbs, B. T., Smith, Janet M. and Walker, A. W. (1977). Enzymic determination of plasma cholesterol on discrete automatic analysers. *Clin. Chim. Acta* **79**, 391-397.

[12] Rubenstein, K. E., Schneider, R. S. and Ullman E. F. (1972). Homogeneous Enzyme Immunoassay. A new immunochemical technique. *Biochem. Biophys. Res. Commun.* **47**, 846-851.

### An evaluation of the Chemispek multichannel analyser

**R.W. Logan*, A.K. Tweedie, Evelyn M. Aitchison and Elizabeth C. Jamieson**

*Department of Biochemistry, Yorkhill Children's and Maternity Hospitals, Glasgow, UK.*

### Description

The Chemispek is a multichannel analyser of the continuous flow type with a microprocessor-based system for collection and presentation of results. Modular in design, the equipment is similar in many respects to the earlier Chromaspek amino acid analyser produced by the same company (Rank Hilger, Margate).

At present, up to twelve simultaneous channels may be operated, chosen from a range of seventeen chemistries.

An eight channel configuration was chosen as being most suitable for the requirements of this department providing the following analyses: sodium, potassium, chloride, carbon dioxide, urea, creatinine, calcium and phosphate.

The instrument is supplied complete with a mounting stand although the individual modules may be bench mounted. The dimensions are length, 2.5 m; depth 0.8 m and height, 1.5 m. An additional stand carrying a chart recorder and microprocessor measures 0.8 m in length, 0.8 m in depth and 1 m in height. There is also a free standing matrix printer of similar dimensions to the smaller stand.

**Correspondence should be addressed to Dr. R.W. Logan, Consultant Medical Biochemist at the above address.*

The instrument is designed to be operated at sampling speeds of up to 120/h depending upon the chemistries involved. As no system of positive sample identification is provided, it is necessary to record the exact sequence of analysis of the specimens in the trays. The sampler module provides for switch-selectable sampling rates covering a wide range of settings. The sample to wash ratio is variable and there is a facility to introduce an air bubble between samples.

Reagents are distributed to each twin channel chemistry module by means of four-speed peristaltic pumps. Wash valves provide the means by which all or individual channels may be switched to wash water. The frequency of air injection to the flowing reagent stream may be altered by thumbscrews mounted on the introduction blocks. The fast speed setting of the pumps allows for rapid filling of the system with reagent or fast wash out with water, while the slowest pump speed setting allows the machine to be kept in a state of readiness over long periods while using a minimum of reagent.

Heating baths, dialysers, coils and connecting tubing are mounted on removable plastic trays. Effluent is channelled away through a series of interconnected drains which may be flushed with water to dilute the waste. A floor level drain would be a requirement for permanent siting of the instrument.

The equipment may be operated from one 13 A fused mains outlet although the microprocessor should preferably be linked to a separate outlet. Interconnective electrical cabling is conveniently channelled through a plastic trough at the rear of the machine.

The twin channel photometers are fitted with a quartz iodine light source and interference filters for wavelength selection. Debubbling of the segmented flow stream before colorimetric measurement is avoided as the photometers have an associated bubble decoding circuit.

A curve regeneration type of response facility with variable settings is provided to improve peak quality and reduce carryover effect.

Sodium and potassium measurements are performed on an Instrumentation Laboratories Model 543 flame unit and a small compressor provides the necessary air supply. A source of propane gas must be provided and suitable connections are available for cylinders.

A digital to analogue converter unit is provided to link the flame photometer to a dual channel chart recorder. The signals from the other six chemistries on the instrument are fed to a multipen recorder to provide a permanent trace of analyses performed.

Control of the instrument and collection and printing of results are by means of a microprocessor fitted with multiplexing facilities for data capture and a dot matrix printer for output. The program, held in read only memory, captures incoming data and stores it in read and modify memory. Keyboard commands set various parameters within the program, eg, the number of decimal places to be printed for each chemistry, the order of printout and identification data for each sample or standard run.

The system is designed to be flexible and each channel may be individually programmed for a wide range of parameters.

Drift correction may be selected and a maximum limit set beyond which results are displayed uncorrected and flagged.

Carryover correction is another option and the normal range outside which results are marked may also be set. Facilities are available for blank channel data to be subtracted where required.

The delay time, ie, the elapsed time between sampling and the appearance of peaks on the recorders, is alterable for each chemistry, and the amount of variation allowed in times between peak maxima may be set so that non-standard shaped peaks can be indicated.

Flexibility in calculation of standard curves is provided with linear, quadratic and cubic fits available on an individual channel basis. A zero point may be included and the number of standard points per chemistry can be selected.

The front panel of the microprocessor is fitted with an LED display which indicates the status of each chemistry by means of a number. On/off switches are fitted so that any channel may be taken 'offline'. An attention switch allows transfer of control to the printer keyboard and a marker switch enables significant events to be indicated on the printout. Once set up, the selected parameters may be stored in a cassette recorder and if the microprocessor has to be switched off the settings may be recalled from tape by means of a keyboard command.

A hard copy is provided by means of a dot matrix printer which presents the results in horizontal format. Cup number, run number and other identifying data are also printed, and different error conditions as seen by the software are indicated by the printing of coded characters under the results.

**Table 1. Total carryover**

| Channel | Units | A3 | B3 | % Interaction | |
|---|---|---|---|---|---|
| | | | | Uncorrected | Corrected |
| Sodium | mmol/l | 160.80 | 104.80 | 0 | -0.9 |
| Potassium | mmol/l | 8.05 | 1.91 | 0.1 | 0 |
| Chloride | mmol/l | 117.70 | 82.90 | 3.8 | 0.4 |
| Carbon dioxide | mmol/l | 37.20 | 17.70 | 2.4 | 0.5 |
| Urea | mmol/l | 30.60 | 4.78 | 1.8 | 0.8 |
| Creatinine | μmol/l | 502.00 | 124.20 | 5.2 | 1.8 |
| Calcium | mmol/l | 3.13 | 1.89 | 11.8 | 2.1 |
| Phosphate | mmol/l | 4.90 | 0.59 | 2.9 | 0.7 |

### Analytical methods

A brief indication of each methodology is as follows :

| | |
|---|---|
| Sodium/potassium | IL Flame photometer<br>Internal lithium standard - no dialysis |
| Chloride | Mercuric thiocyanate/ferric nitrate [1] |
| Carbon dioxide | Gas dialysis - cresol red |
| Urea | Diacetyl monoxime [2] |
| Creatinine | Alkaline picrate [3] |
| Calcium | Cresolphthalein complexone [4] |
| Phosphate | Molybdo-vanadate [5] |

### Materials and methods

### Reagents

Working solutions supplied with the instrument were from BDH Chemicals, Poole, Dorset. During the period of the evaluation, reagents supplied by the Boehringer Corporation (London) Ltd, were also used. Reagents are supplied in 4.5 l containers which store conveniently on the shelving provided under the analytical modules.

### Standardisation and operation

Prior to the analysis of specimens a set of standards either aqueous or serum based, conforming to an optional predetermined format, has to be run in order to enable the microprocessor program to convert voltage readings thereafter to concentration values based on the calibration curve generated for each channel. The number of standards is flexible up to a maximum of seven and they may be run in any order. This standard table is normally run at the start of each day and may be repeated if instrumental drift is indicated or if any alterations are made to the sampling speed or other similar parameters.

The particular configuration chosen for the instrument under evaluation presented a more complex standardisation problem than that normally encountered with the more common six channel system. Because of the fact that the possibility existed to extend the instrument to handle twelve analytical channels simultaneously, it was decided to employ a mixture of aqueous and protein-containing solutions for the calibration process. The standardisation was normally repeated until a satisfactory value for drift was obtained for all analytical channels.

Samples are analysed in batches of eight interspersed with duplicate drift cups to enable the drift value to be updated by the computer program. The sample tray contains space for 60 cups and as the first and last pair are normally occupied by drifts, a total of 46 specimens may be analysed in each run. It is not possible to add a second tray immediately at the end of the first and the number of specimens should be known in advance although the software makes provision for additions provided that the analysis is terminated by a double pair of drift specimens.

### Procedure

Evaluation procedures were based on a recommended scheme [6] and no attempt was made to modify the equipment in any way during the trial.

Patient samples and quality control materials covering the wide range of concentrations likely to be encountered in routine analysis were employed in the method comparison

**Table 2. Within-batch precision at three concentrations**

| Channel | | Low | Medium | High |
|---|---|---|---|---|
| Sodium mmol/l | Mean | 119.00 | 140.90 | 148.00 |
| | SD | 0.59 | 0.88 | 0.97 |
| | CV (%) | 0.50 | 0.63 | 0.65 |
| Potassium mmol/l | Mean | 2.99 | 5.00 | 6.82 |
| | SD | 0.02 | 0.03 | 0.05 |
| | CV (%) | 0.49 | 0.50 | 0.77 |
| Chloride mmol/l | Mean | 82.00 | 107.90 | 119.00 |
| | SD | 0.82 | 1.26 | 1.01 |
| | CV (%) | 1.00 | 1.17 | 0.85 |
| Carbon dioxide mmol/l | Mean | 17.39 | 27.28 | 36.79 |
| | SD | 0.19 | 0.44 | 0.50 |
| | CV (%) | 1.07 | 1.61 | 1.36 |
| Urea mmol/l | Mean | 4.39 | 21.64 | 29.21 |
| | SD | 0.06 | 0.42 | 0.19 |
| | CV (%) | 1.40 | 1.93 | 0.66 |
| Creatinine μmol/l | Mean | 120.00 | 331.60 | 520.20 |
| | SD | 1.51 | 6.17 | 11.02 |
| | CV (%) | 1.26 | 1.86 | 2.12 |
| Calcium mmol/l | Mean | 1.88 | 2.86 | 3.10 |
| | SD | 0.03 | 0.08 | 0.03 |
| | CV (%) | 1.70 | 2.76 | 1.09 |
| Phosphate mmol/l | Mean | 0.67 | 1.52 | 2.17 |
| | SD | 0.01 | 0.02 | 0.02 |
| | CV (%) | 2.03 | 1.26 | 0.87 |

**Table 3. Between-batch precision at three concentrations**

| Channel | | Low | Medium | High |
|---|---|---|---|---|
| Sodium mmol/l | Mean | 116.90 | 134.20 | 145.70 |
| | SD | 3.59 | 2.69 | 4.61 |
| | CV(%) | 3.07 | 2.01 | 3.16 |
| Potassium mmol/l | Mean | 2.95 | 4.47 | 6.67 |
| | SD | 0.08 | 0.09 | 0.19 |
| | CV(%) | 2.68 | 2.09 | 2.88 |
| Chloride mmol/l | Mean | 80.30 | 93.10 | 117.00. |
| | SD | 3.77 | 2.51 | 3.73 |
| | CV(%) | 4.70 | 2.69 | 3.19 |
| Carbon dioxide mmol/l | Mean | 16.79 | 21.42 | 34.06 |
| | SD | 1.25 | 0.77 | 2.16 |
| | CV(%) | 7.46 | 3.58 | 6.34 |
| Urea mmol/l | Mean | 4.43 | 10.68 | 29.88 |
| | SD | 0.33 | 0.42 | 1.25 |
| | CV(%) | 7.34 | 3.96 | 4.19 |
| Creatinine μmol/l | Mean | 127.20 | 149.00 | 519.50 |
| | SD | 4.94 | 3.85 | 28.04 |
| | CV(%) | 3.88 | 2.59 | 5.40 |
| Calcium mmol/l | Mean | 1.82 | 2.52 | 3.06 |
| | SD | 0.07 | 0.10 | 0.11 |
| | CV(%) | 3.69 | 3.76 | 3.53 |
| Phosphate mmol/l | Mean | 0.71 | 1.49 | 2.16 |
| | SD | 0.11 | 0.08 | 0.09 |
| | CV(%) | 5.31 | 5.11 | 4.10 |

**Table 4. Linearity of response**

| Channel | Units | Range tested | Range linear |
|---|---|---|---|
| Sodium | mmol/l | 100 - 200 | 100 - 200 |
| Potassium | mmol/l | 0 - 10 | 0 - 10 |
| Chloride | mmol/l | 70 - 130 | 70 - 130 |
| Carbon dioxide | mmol/l | 0 - 40 | 5 - 40 |
| Urea | mmol/l | 0 - 50 | 0 - 50 |
| Creatinine | μmol/l | 0 - 800 | 0 - 800 |
| Calcium | mmol/l | 0 - 5 | 0 - 4 |
| Phosphate | mmol/l | 0 - 5 | 0 - 5 |

survey. Similar analytical methods using a flame photometer and continuous flow automated equipment were used for sodium, potassium, chloride, urea, carbon dioxide and creatinine estimations. The calcium and phosphate analyses were performed on a Greiner selective analyser using a cresolphthalein method for calcium and a procedure forming a complex of phospho-molybdate with malachite green for the phosphate estimation.

### Overall performance

As a preliminary check on the precision of machine performance three groups of eight samples were analysed for all channels after satisfactory standardisation had been achieved. The results indicated no obvious problems.

### Sample size

The particular configuration of analyses performed upon the machine under test required a sample volume of 260 μl averaged over a representative period of time. This is a convenient volume for the 0.5 ml autoanalysis cups commonly in use in paediatric biochemistry laboratories.

### Total carryover

Sample interaction was measured by analysis of ten alternating groups containing three specimens of elevated and three of low concentration ie specimens A1, A2, A3 contained a high level and were followed by specimens B1, B2, B3 containing a low level of concentration for all channels.

The instrument is provided with a selectable option which enables carryover to be measured during the standard table run which precedes the sample run by averaging the differences between the first and second of the standard pairs and applying the calculated values to the sample run. The effectiveness of this procedure was assessed by performing the above experiment with and without carryover correction. The results, showing average A3 and B3 values, and percentage interaction both corrected and uncorrected are illustrated in Table 1.

### Precision

Within-batch precision was measured at high, low and mid range levels of concentration by analysis of one complete tray of replicates. Samples which were stored deep frozen were employed for assessment of between batch precision which took place over a period of several months. Table 2 lists the results for within-batch and Table 3 the between-batch figures.

### Linearity

The standardisation procedure allows for the fitting of a curve to a channel producing a non-linear response. After a preliminary assessment it was decided to employ this facility on measurements from the carbon dioxide channel. Serum and aqueous preparations were then analysed to assess the linearity of each channel over the range likely to be required in normal use. Table 4 lists the range investigated and the limits of linear response.

### Accuracy of analysis

Commercial control sera were analysed repeatedly over a period of several months and the average results compared with the manufacturer's target values for the appropriate methodologies (Table 5).

Method comparison studies were performed by analysing at least 100 patient samples by both the Chemispek and the current methods used within this hospital district. The results were subjected to regression analysis and are presented in Table 6.

### Results and discussion

### Precision

Preliminary testing indicated that the instrument was capable of good reproducibility within a given batch of analyses. This was borne out by the results obtained from within-batch reproducibility studies (Table 2). Overall, the results show a remarkably low coefficient of variation at low, medium and high levels of concentration for all channels.

The poor figures obtained for between-batch reproducibility (Table 3) reflect some of the problems experienced during operation of the instrument over a long period of time. These problems are more fully discussed below.

### Sample interaction

For practical purposes carryover was undetectable on the sodium and potassium channels (Table 1). Despite the high sampling rate (120 per hour) interaction is relatively low on all but the creatinine and calcium channels. The use of the carryover correction provided by the software helped to reduce the effect on results on all affected channels with a tendency to overcorrect where there was no apparent interference as in the case of the sodium estimation. It is understood that a future version of the software will incorporate an improved means of measuring carryover based on the method described by Broughton *et al* [7].

An alternative means of reducing the carryover effect is by employment of the response facility provided on the photometers. This method was found to be less effective overall in reducing sample interaction but it produced considerable improvement in peak quality and resolution of shoulder peaks.

### Linearity

Linearity of response for all channels covered the useful range likely to be encountered in routine analysis and the vast majority of specimens could safely be analysed without prior dilution. Table 4 indicates slight deviations from linearity at the bottom end of the carbon dioxide range. This could probably be eliminated by the use of additional standards at that level although the benefit obtained would seem to be of doubtful clinical significance.

### Accuracy of analysis

Values for the five quality control specimens used gave good agreement between found values on the Chemispek and target values on all channels with the exception of sodium where the values found were consistently lower. This may be a reflection of the limitations of the standardisation process where only seven separate standards must provide for the full ranges encountered over eight different chemistries. Another contributing factor may be the hydraulic effects of the closed sampling system where the sodium/potassium analysis is last in the sequence. It was noted that the flame sometimes drifted considerably during the working day, although not sufficiently to exceed the limits for drift set with the software.

In the analysis of patient samples (Table 6), poor correlation of sodium and potassium is evident and again may reflect the standardisation problems mentioned above.

The scatter of results obtained from the carbon dioxide comparisons is probably due in part to loss occurring as a result of differences in time of analysis between the methods, the' problems of storage and the transfer of very small specimens. Urea and creatinine results on patient comparisons gave excellent correlation reflecting the trouble free performance of these channels.

The disappointing results obtained for chloride may again be due to inadequacies in the standardisation process. A frequent problem encountered on the calcium channel was noise, the cause of which was difficult to attribute to any particular condition, and it is, therefore, hardly surprising that the correlation of patient results with the standard method is less than satisfactory. Reasonable comparisons were obtained with the phosphate channel despite the differences in methodology.

In the course of performing the patient comparison study, haemolysed, lipaemic and jaundiced samples were analysed, and no significant difference in results was observed between the methods.

**Table 6. Comparison of patient results**

| Channel | Range of values | Slope | Y-Intercept | Correlation coefficient |
|---|---|---|---|---|
| Sodium | 110 - 150 | 0.92 | 10.80 | 0.956 |
| Potassium | 2 - 7 | 0.97 | 0.19 | 0.968 |
| Chloride | 75 - 130 | 0.91 | 9.80 | 0.933 |
| Carbon dioxide | 10 - 40 | 0.99 | 0.95 | 0.910 |
| Urea | 1 - 50 | 1.03 | −0.29 | 0.997 |
| Creatinine | 10 - 650 | 1.04 | 7.90 | 0.995 |
| Calcium | 1.5 - 3.5 | 0.90 | 0.22 | 0.926 |
| Phosphate | 0.5 - 3.5 | 0.95 | 0.05 | 0.939 |

**Table 5. Control comparisons**

| Channel | | A | B | C | D | E | Average |
|---|---|---|---|---|---|---|---|
| Sodium | MV | 141.00 | 148.00 | 132.00 | 147.00 | 155.00 | 144.60 |
| | CSPEK | 137.90 | 146.10 | 127.30 | 142.30 | 152.40 | 141.20 |
| Potassium | MV | 4.70 | 5.50 | 4.00 | 5.70 | 6.40 | 5.26 |
| | CSPEK | 4.61 | 5.38 | 4.01 | 5.43 | 6.33 | 5.15 |
| Chloride | MV | 93.00 | 100.00 | 95.00 | 106.00 | 105.00 | 99.80 |
| | CSPEK | 93.40 | 99.60 | 94.30 | 105.70 | 107.20 | 100.00 |
| Carbon dioxide | MV | 18.80 | 23.00 | 15.00 | 22.00 | 27.00 | 21.16 |
| | CSPEK | 20.39 | 24.53 | 17.60 | 23.47 | 27.46 | 22.69 |
| Urea | MV | 10.40 | 30.50 | 15.70 | 17.70 | 29.30 | 20.70 |
| | CSPEK | 10.67 | 30.29 | 16.01 | 18.17 | 29.47 | 20.92 |
| Creatinine | MV | 174.00 | 358.00 | 669.00 | 369.00 | 719.00 | 457.80 |
| | CSPEK | 164.60 | 341.10 | 645.10 | 360.70 | 696.20 | 441.50 |
| Calcium | MV | 2.39 | 2.90 | 2.16 | 2.67 | 3.00 | 2.62 |
| | CSPEK | 2.39 | 2.97 | 2.16 | 2.67 | 3.02 | 2.64 |
| Phosphate | MV | 1.67 | 1.15 | 2.16 | 1.70 | 2.13 | 1.72 |
| | CSPEK | 1.66 | 1.16 | 2.06 | 1.59 | 2.08 | 1.71 |

MV = Manufacturer's value

### Limits of detection

In order to determine whether the peak recognition capabilities of the instrument were adequate to detect the very low levels occasionally found in patient samples, a series of dilutions were prepared and the results were examined with particular reference to the urea and creatinine channels. It was found that the analyser could resolve peaks and provide accurate measurements down to creatinine concentrations of 10 μmol/l and urea concentrations of 1 mmol/l.

### Maintenance

Apart from routine disinfection of the sampling systems after processing patient samples, very little maintenance is required. Pump tubes need changing approximately every two weeks and dialyser membranes on a weekly basis. The reagent valves had to be examined daily as they had a tendency to block readily. Small plasma samples are the most common type of specimen in use in this laboratory and blockages of narrow bore sampling tubes are not uncommon. It was found necessary to check all potential blockage points frequently as the flame unit in particular seemed to suffer in this respect.

The multipen chart recorder is very compact in design and capping the pens after use to prevent them drying out proved to be rather difficult due to the very limited access.

### Instruction manuals

A comprehensive operator's manual covers the analytical aspects of the instrument. This manual deals adequately with the installation procedure and the sections covering the operation of the equipment give all the information necessary for it to be run. The information is, however, scattered in various sections of the manual and was found to be difficult to collate. There is a fault-findng guide and an extensive list of spare parts together with part numbers.

A brief description of the daily set-up procedure would be a helpful addition.

A preliminary booklet was supplied on the use of the microprocessor. This was found to be difficult to follow and in places there were errors which could have resulted in wrong information being keyed into the computer. The operator has to read and thoroughly digest the information before he could attempt to operate the microprocessor. Since the computer offers so many machine operation options a more detailed description should be given to enable the correct choice to be made.

### Running costs

A rough estimate of running costs is given below. This is based on 250 samples per day over a working year of 260 days. Staffing requirements are difficult to estimate as the instrument was not operated on a routine basis but from previous experience a figure of 1.5 staff would seem to be realistic.

| | Cost per sample (p) |
|---|---|
| Depreciation | 8.0 |
| Reagents | 0.7 |
| Reference sera | 3.4 |
| Consumables | 6.1 |
| 1.5 MLSO staff | 11.6 |
| Total | 29.8 |

The depreciation was calculated on the basis of a capital cost of £35,000 for the instrument and an expected life of about ten years. The sum shown actually includes an estimate of the maintenance costs for the instrument. A full service contract for comparable instruments ranges between 8-10% of the capital cost per annum. The salary of a Medical Laboratory Scientific Officer (MLSO) was taken as £5000 per annum.

### Record of machine performance

Shortly after installation, all the three-inch dialysers were found to be warped and leaking. This problem was rapidly dealt with by the company who provided new dialysers with reinforced back plates which have proved satisfactory. A number of small connective pieces came apart due apparently to the wrong type of adhesive having been employed.

One of the pumps had to be replaced as it was found to slip out of gear and stop pumping.

The wash valves appear to be prone to blockage on the calcium channel in particular, hardening of the silicone tubing caused the valve to become inoperative. Bypassing the reagent valves was often the only means of obtaining a stable baseline.

Apart from the relatively minor problems mentioned above, the instrument suffered from considerable 'down time' due to problems with the microprocessor computer program. The program faults resulted in corruption of some registers causing bizarre effects, eg complete loss of printout, duplication of printout and, on two occasions, output of results several days after the samples had been aspirated. Occasionally the sample turntable would stop after the first specimen had been sampled and upon inspection it was found that all the set-up parameters in the program had reverted to their default values.

In the early stages of the evaluation, tracer peaks on the phosphate channel often triggered the flashing of the LED display which is meant to draw the operator's attention to a problem condition when no such condition seemed to exist. The program was amended to eliminate this. During the analysis of samples whose concentration on some channels was close to zero, the program sometimes printed asterisks and 'H' flags meant to indicate values above the normal range, instead of results. An alteration was made to the software which eliminated this fault. The problem was a software fault which allowed carryover correction during the running of a standard table instead of ignoring this facility.

A suggestion was made by the manufacturers that incorrect use of terminators on inspection or alteration of the set-up parameters was causing corruption of the data held in store. This corruption is invisible to the operators and could result in unpredictable results.

Due in part to the unusual nature of the circumstances causing the difficulties (they seem to be related to the fact that our instrument was the only eight channel unit in use) and the intermittent nature of the faults, it took about five months before the major software problem was identified and rectified.

During this period a constant voltage transformer fitted with noise suppression was attached as it was thought that mains-borne interference could be causing some of the effects. A subsequent investigation of the electrical supply failed to confirm this.

The microprocessor unit itself was replaced three times and the programmable read-only memories (proms) were updated twice during this time in an effort to resolve the problems.

The main difficulty now appears to have been overcome although the instrument would have to be run for an extended period to be certain of this. The company expended considerable time and effort in order to resolve the problems in the software, although from the amount of time required, this would seem to have been an area of weakness.

### General

Operation of the analytical side of the equipment is fast and simple for staff familiar with continuous flow technology. The modular layout and well designed benching facilitates access to controls and reagent supplies.

Set up of the parameters used by the computer program is rather more difficult and the use of different terminators for input, inspection and alteration of the data makes the process confusing and error prone, particularly for staff unused to computer input. Once entered, however, the parameters do not normally have to be altered and may be stored on a cassette recorder and recalled when necessary. This is one area where the software could be considerably improved.

Blockage of sample lines due to fibrin clots is not uncommon where the very small heparinised specimens used in paediatric work form a large part of the workload and this led to some operating problems. The sample system on the Chemispek is a closed one in that there is no predilution circuit and all the aspirated specimen is consumed, none being left to flow to waste. The effect of this is that a blockage at the flame unit, the last in sequence, causes other channels to be affected.

Consumption of reagents was extremely low, even with the instrument operating for the bulk of the working day. In routine use it would be prudent to replace some of the 4.5 l containers with smaller capacity bottles as some of the reagents would deteriorate before being used.

The useful life of pump tubes seemed to be somewhat shorter on the Chemispek than that normally associated with other continuous flow equipment.

The need to process standards and samples in separate batches is a limitation where there is a need for emergency analyses out of hours. We understand that a future issue of software will allow a short pattern of standards to be followed immediately by samples. It would be helpful to have the capacity to run a larger number of standards, particularly on machines of more than six channels.

Various error and other conditions are brought to the operator's attention by the printing of coded characters beside the results. We found these 'flags' to be of doubtful value, particularly the one indicating noise during peak measurement. On some occasions, peaks which visually were perfectly normal were flagged as noisy, whereas others giving very distorted tracings were presented without comment.

There is no rapid means of aborting a run short of switching off the microprocessor and reloading the data cassette. If a blockage occurs after some of the samples have been aspirated the specimens have to be removed and reloaded as the sampler carousel cannot be restarted at any but the first position.

As delivered the only means for the recording of results is the printer and no interfacing provision has been made for linkage to laboratory computer systems. The printer output suffers from the fact that communication is one way only, so that if the printer runs out of paper or develops a fault, the microprocessor continues to send results which are then lost.

### Conclusions

For the instrument to be easily incorporated into a routine analytical role a number of improvements would be required in the data processing system provided. In the modern laboratory, computer systems handling the collection and reporting of results play an increasingly important role and new analytical equipment should be provided with proven means of interfacing to such systems.

Accurate linkage of results with patient information is also essential and the lack of patient ID input is a notable omission from the program options provided with the machine.

The wide range of options available for individual channels makes the instrument very flexible but more emphasis should have been placed on solving practical problems such as providing an easy means of restarting an aborted run following a blockage.

Experiences with the microprocessor have not engendered confidence in the data handling side of the equipment, nor in the ability of the company quickly to identify or resolve problems in the software. Nevertheless, from the excellent results obtained from the within-batch precision studies, it is apparent that the instrument using the modified software is capable of producing reproducible results at a very fast sampling rate, and given some redesign of the miroprocessor operations, it has the potential to become a very useful laboratory tool.

ACKNOWLEDGEMENTS
It is a pleasure to acknowledge the willing assitance offered by the manufacturer, Rank Hilger, Westwood, Margate, Kent, and also the financial support afforded by the Department of Trade and Industry which enabled the evaluation to take place.

### Addendum

It should be noted that since completion of the evaluation of the eight channel Chemispek analyser, one of the authors (RWL) has discussed the findings with colleagues in several departments where Chemispek instruments are in routine use. The analysers in question are mainly six channel electrolyte instruments and all appear to be functioning satisfactorily with only minor problems having been encountered by the laboratories concerned.

REFERENCES

[1] Skeggs, L.T. and Hochstrasse, H. (1964), *Clinical Chemistry*, **10**, 285.
[2] Marsh, W.H., Fingerhut, B. and Miller, M. (1965), *Clinical Chemistry*, **11**, 624.
[3] Chasson, A. L., Grady, H. T. and Stanley, M. A., (1961) *American Journal of Clinical Pathology*, **35**, 83.
[4] Gitelman, H.J. (1967), *Annals of Biochemistry*, **18**, 521.
[5] Robinson, R., Roughan, M.E. and Wagstaff, D.F. (1971), *Annals of Clinical Biochemistry*, 8, 168.
[6] Broughton, P. M. G., Gowenlock, A. H., McCormack, J. J. and Neil, D.W. (1974), *Annals of Clinical Biochemistry*, **11**, 207.
[7] Broughton, P. M. G., Buttolph. M. A., Gowenlock, A. H., Neil, D.W. and Skeltenbery, R.G. (1969), *Journal of Clinical Pathology*, **22**, 278.

